# Dermoscopic features of nails in Leprosy patients in a tertiary referral hospital in West Java, Indonesia

**DOI:** 10.1186/s12879-024-09224-0

**Published:** 2024-03-26

**Authors:** Hendra Gunawan, Namira Bening Nurani

**Affiliations:** Department of Dermatology and Venereology, Faculty of Medicine, Universitas Padjadjaran-Dr. Hasan Sadikin Hospital, Bandung, West Java Indonesia

**Keywords:** Dermoscopy, Leprosy, Nail changes

## Abstract

**Introduction:**

Leprosy is a chronic granulomatous infectious disease, mainly affecting the skin and peripheral nerves, caused by the obligate intracellular bacteria *Mycobacterium leprae*. The disease has been discussed in several review articles in recent research, but as far as we know, only a few have addressed the effects of leprosy on nails, especially those who examine the dermoscopic features of nails in leprosy patients.

**Purposes:**

We aimed to document nail changes in leprosy patients and identify any particular findings through dermoscopic examination.

**Method:**

This was an observational study conducted in the Dermatology and Venereology Clinic of Hasan Sadikin Hospital, West Java, Indonesia, from March 2023 through May 2023. All patients have established cases of leprosy, and the diagnosis is based on clinical and bacteriological examinations. Recruitment was done through total sampling. Dermoscopic examination of all fingernails and toenails was performed at 10x magnification using a handheld dermatoscope (Heine DELTA 20 T Dermatoscope) in polarized mode without the linkage fluid to document the dermoscopic features.

**Result:**

Of a total of 19 patients, 15 had nail changes due to leprosy. Out of 15 patients, 13 patients were male. Patients below 25 years old had more nail changes. Most of the patients had a duration of disease greater than two years. Both fingers and toes were involved in nine patients. In this study, the most common dermoscopic feature found was the longitudinal ridge. Other dermoscopic features found in this study were transverse lines, onycholysis, longitudinal melanonychia, leukonychia, subungual hemorrhage, subungual hyperkeratosis, anonychia, and onychorrexis.

**Conclusion:**

Nail changes are found in leprosy patients and have a wide variety of clinical appearances. A dermoscopy should be performed to assess nail changes in leprosy.

## Introduction

Leprosy is a chronic granulomatous infectious disease, mainly affecting the skin and peripheral nerves, caused by the obligate intracellular bacteria *Mycobacterium leprae (M. leprae)* [1, 2]. Leprosy is still a health problem in several developing countries. In 2022, Indonesia still ranks third in the number of new leprosy cases in the world after India and Brazil, with 12,441 cases out of 174,068 new cases worldwide [3].

The nerves and skin are the primary targets of *M. leprae* infection, but other organs and tissues (eyes, joints, testis, and lymph nodes) can also be affected [4]. The development of functional limitations and a partial or complete disability can directly affect the patient’s quality of life [5]. The disease has been discussed in several review articles in recent research, but, as far as we know, only a few articles have addressed the effects of leprosy on nails [6–9].

The incidence of nail changes was reported to be 64% in the only published study involving a large number of leprosy patients [8]. In leprosy, the cause of changes is frequently multifactorial. Neuropathy is the most common cause of nail abnormalities in leprosy, however other factors such as recurrent trauma, vascular impairment, infections, or negative therapeutic side effects can also impact the nail plate, matrix, bed, and periungual skin folds [7, 8].

Nail dermoscopy was used to evaluate nail pigmentation, but its use has expanded to diagnose nail disorders. Numerous nail signs can be magnified with dermoscopy and combined with a clinical examination to make a diagnosis [13]. Previous research on nail changes in leprosy found no specific manifestation of the disease but did find a higher frequency of nail changes in those who were affected [6–9]. To our knowledge, no research has been conducted on the dermoscopy examination of nails in leprosy patients in Indonesia. This study aimed to document nail changes in leprosy patients and identify any findings with dermoscopic examination.

## Materials and methods

This observational study was conducted in the Dermatology and Venereology Clinic of Hasan Sadikin Hospital, West Java, Indonesia. Every patient has confirmed leprosy cases, and the diagnosis was based on clinical and bacteriological examinations. They were recruited for dermoscopic evaluation from March 2023 through May 2023. Recruitment was done through total sampling. All patients received multidrug therapy (MDT) for leprosy. A direct microscopic examination with 10% potassium hydroxide was performed on patients suspected of having onychomycosis. Patients of all age groups were included in the study; patients who had diseases known to cause nail changes (psoriasis, lichen planus, and alopecia areata) and those with predisposing factors that could produce peripheral neuropathy (diabetes mellitus) were excluded from the study. All patients were subjected to a clinical examination for leprosy and a dermoscopic examination of the nails. A handheld dermatoscope (Heine DELTA 20 T Dermatoscope) with a magnification of 10x was used in all cases to examine the nails of the fingers and toes. Images were recorded directly with the dermatoscope’s digital single-lens reflex (DSLR) camera series 1200D and an attachment for the DSLR camera. Polarized modes were used without linkage fluid.

## Results

According to the study’s findings, there were 19 cases of leprosy, but four patients did not show nail abnormalities and hence were not included in the study data. The study included 15 leprosy patients, 13 (13/15) males and 2 (2/15) females. Although the proportion was similar between all age groups, patients below 25 years old had more nail changes. The disease duration in 14 (14/15) patients was below two years, and in one (1/15) patient, it was more than two years. Most of the leprosy patients (9/15) had nail changes in both fingers and toes, while six (6/15) patients had toe involvement only. There were no leprosy patients who had only finger involvement (Table 1).

**Table 1:** Clinical profile of leprosy patients

Group	Frequency (*n* = 15)	Proportion
**Sex**		
Male	13	13/15
Female	2	2/15
**Age**		
< 25 years	6	6/15
25–50 years	4	4/15
> 50 years	5	5/15
**Disease Duration**		
< 2 years	14	14/15
> 2 years	1	1/15
**Finger/Toe Involvement**		
Finger involvement only	0	0/15
Toe involvement only	6	6/15
Finger and toe involvement	9	9/15

**Fig. 1:**
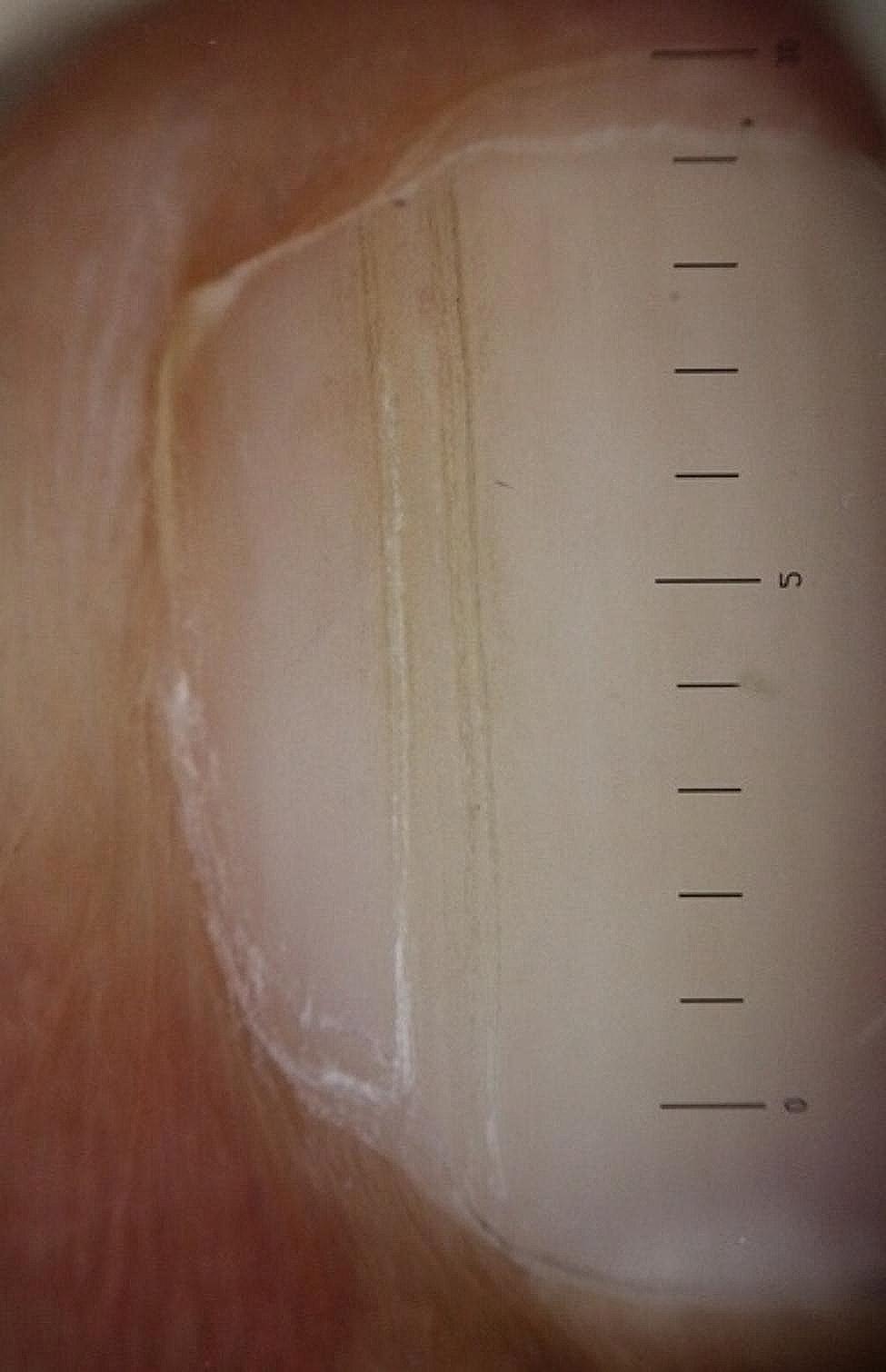
Longitudinal ridge in the nail of a leprosy patient (Heine DELTA 20 T Dermatoscope–polarizing light, ×10)

**Fig. 2:**
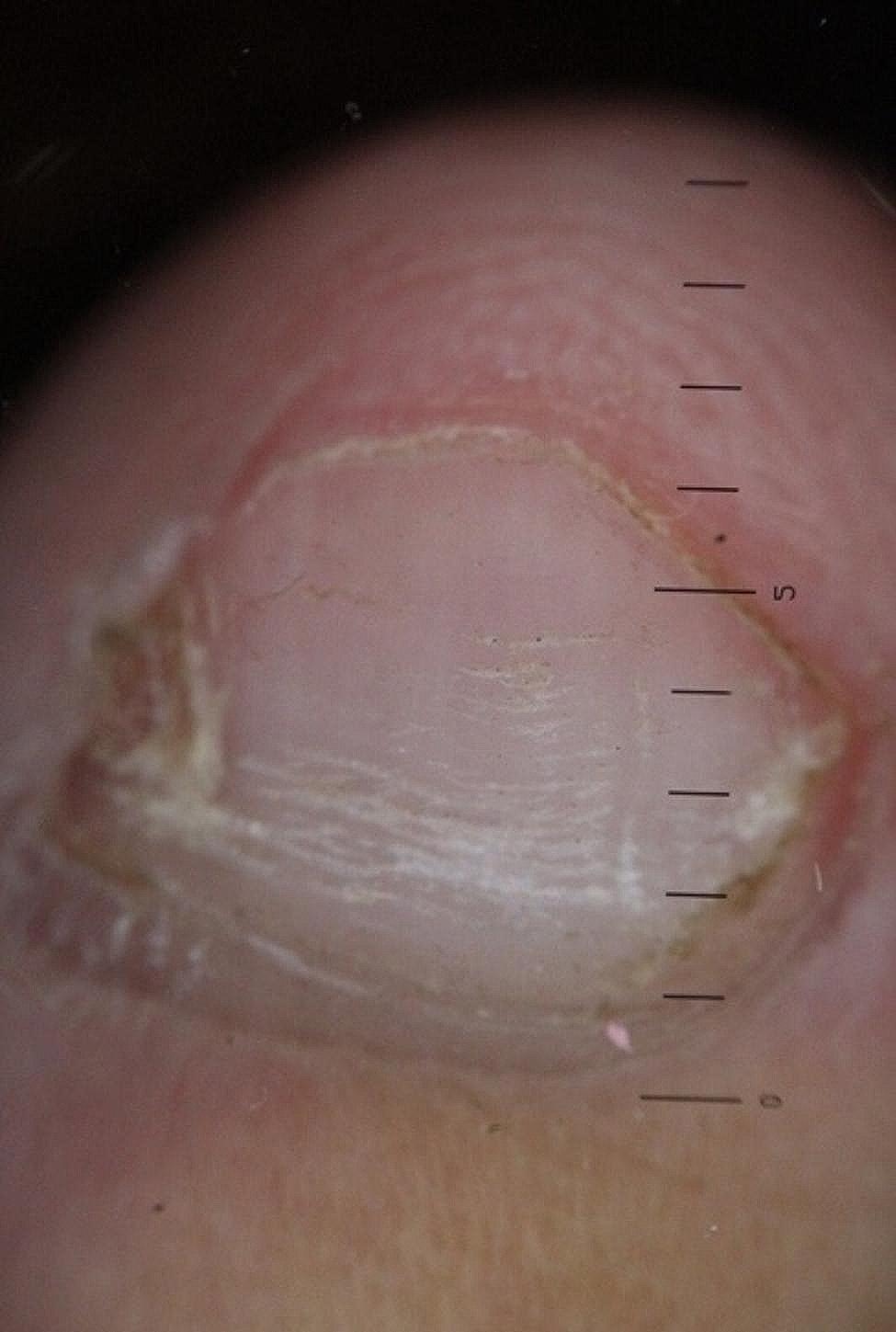
Transverse line in the nail of a leprosy patient (Heine DELTA 20 T Dermatoscope–polarizing light, ×10)

**Fig. 3:**
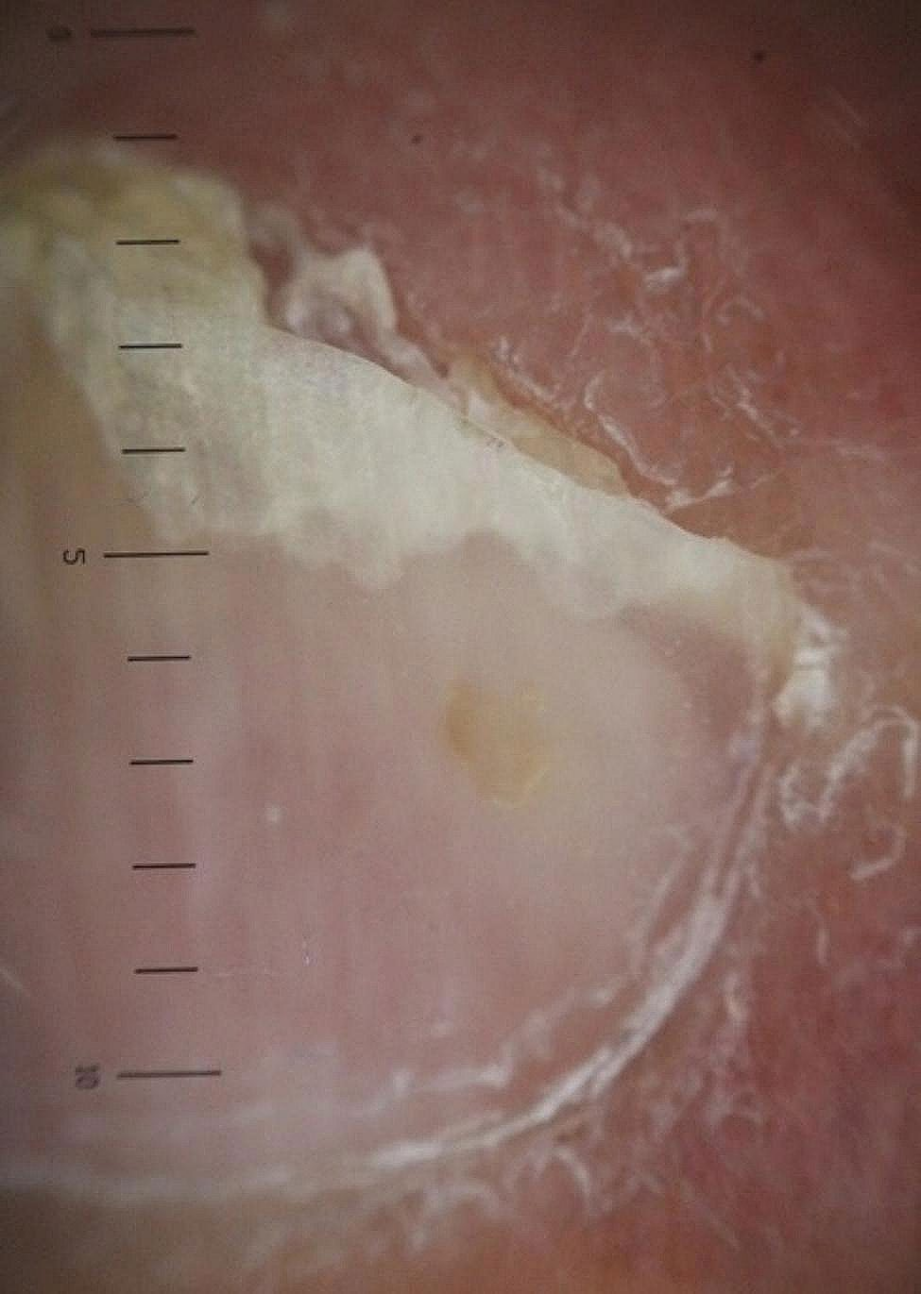
Onycholysis in the nail of a leprosy patient (Heine DELTA 20 T Dermatoscope–polarizing light, ×10)

**Fig. 4:**
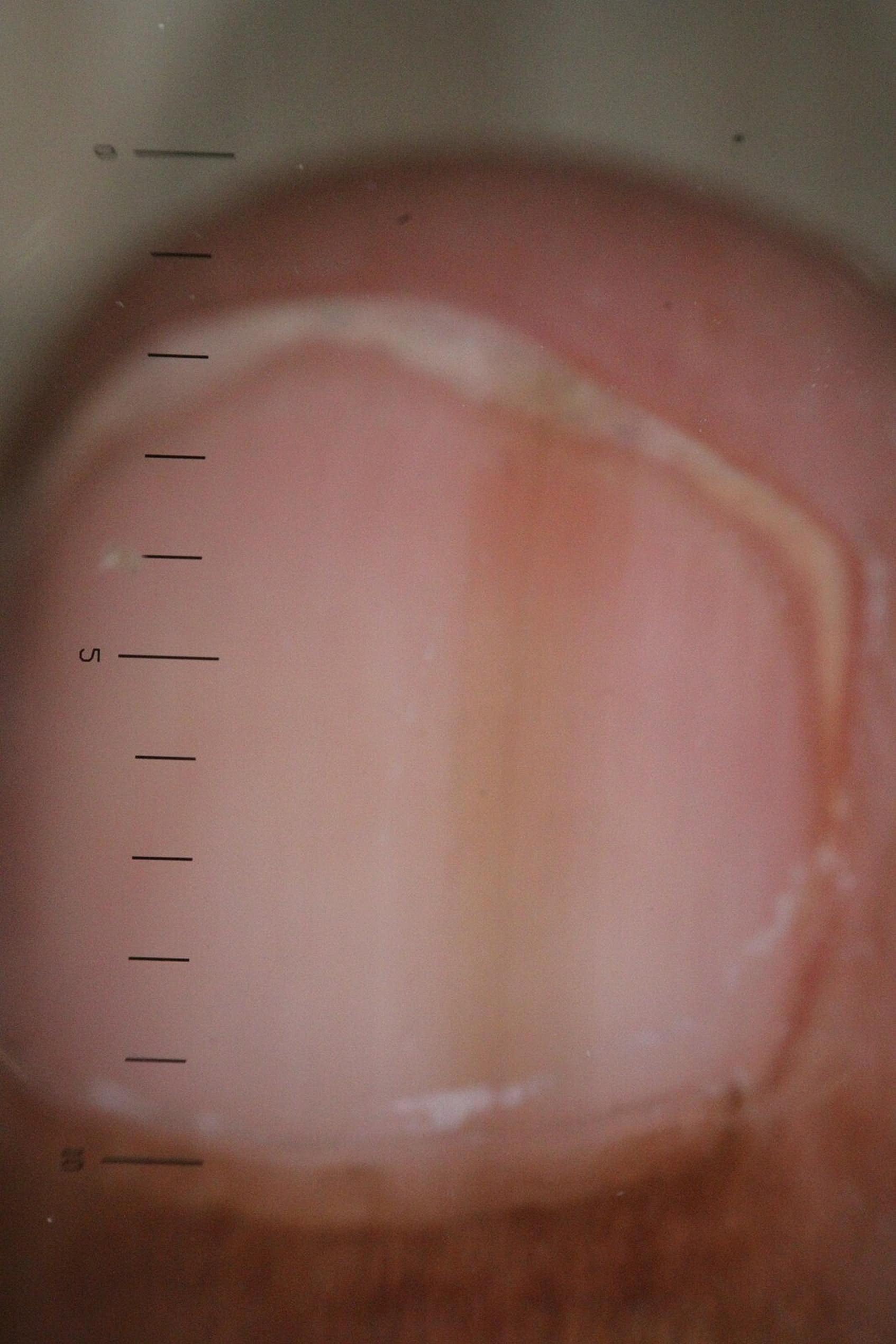
Longitudinal melanonychia in the nail of a leprosy patient (Heine DELTA 20 T Dermatoscope–polarizing light, ×10)

**Fig. 5:**
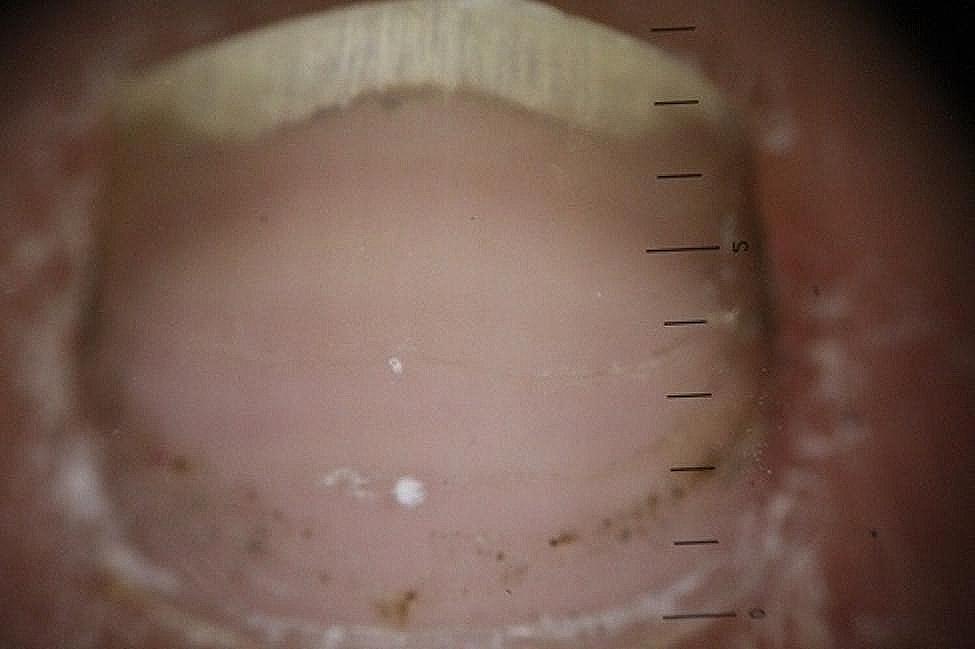
Leuconychia in the nail of a leprosy patient (Heine DELTA 20 T Dermatoscope–polarizing light, ×10)

**Fig. 6:**
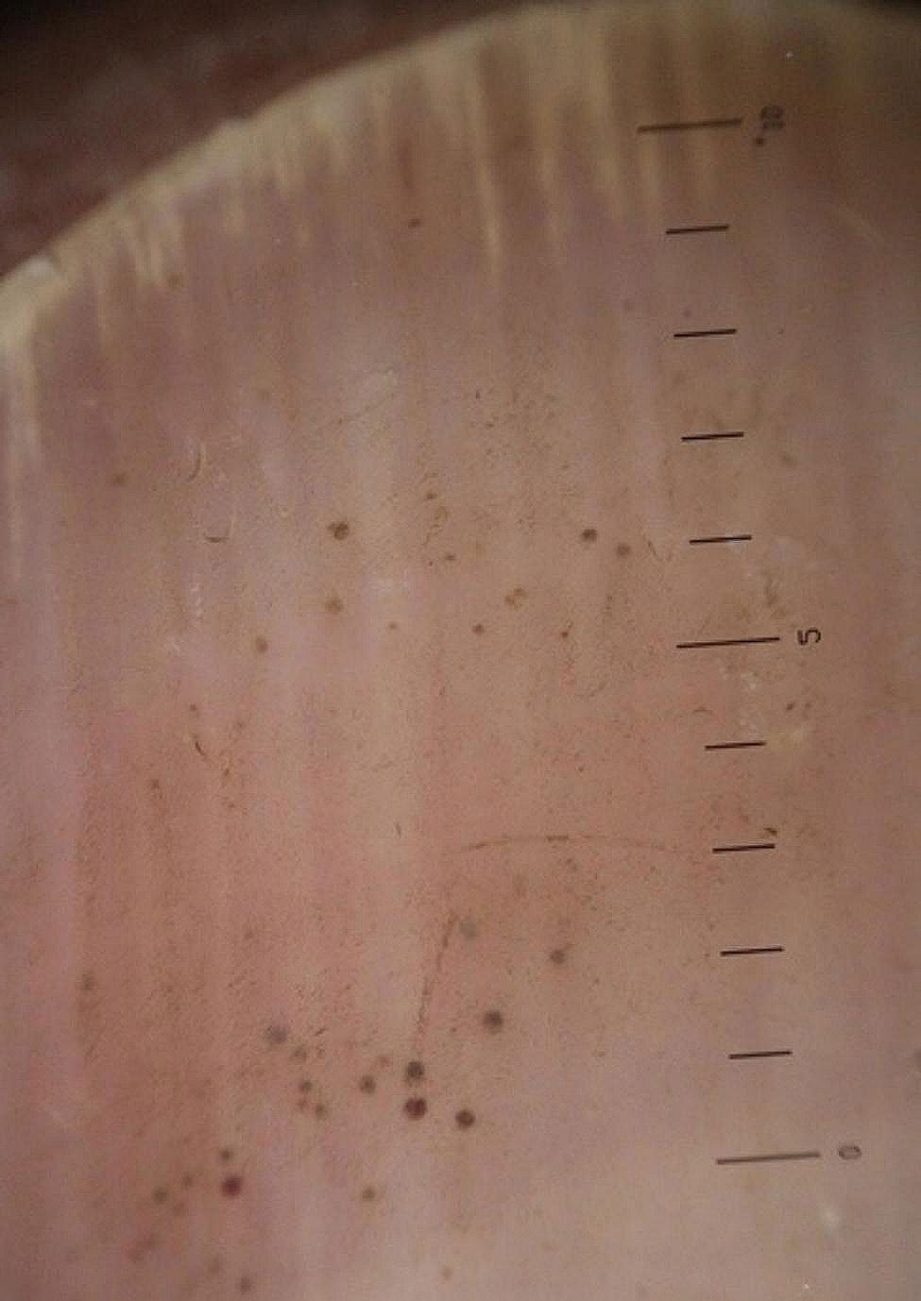
Subungual haemorrhage in the nail of a leprosy patient (Heine DELTA 20 T Dermatoscope–polarizing light, ×10)

**Fig. 7:**
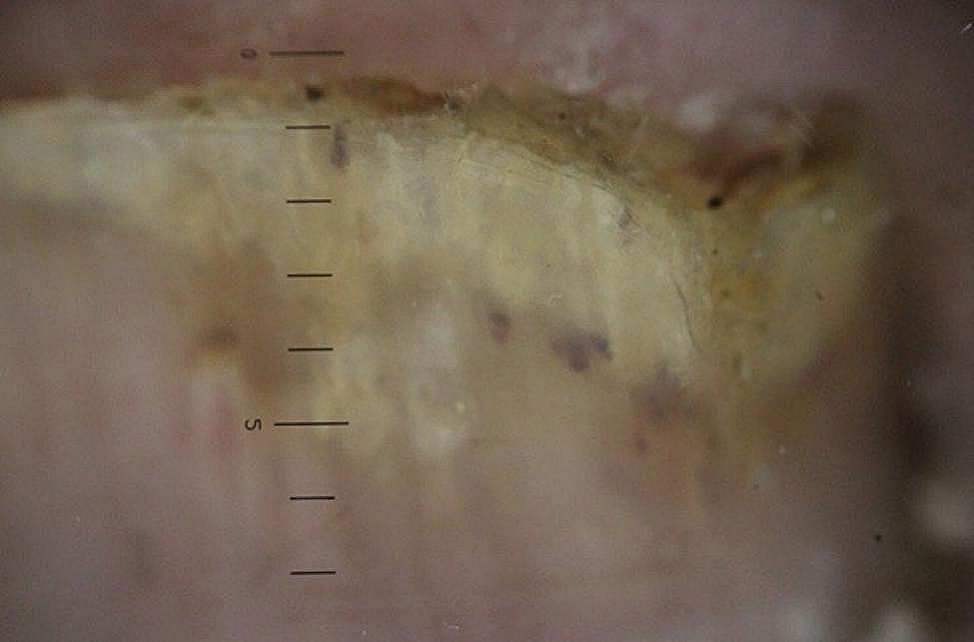
Subungual hyperkeratosis in the nail of a leprosy patient (Heine DELTA 20 T Dermatoscope–polarizing light, ×10)

**Fig. 8:**
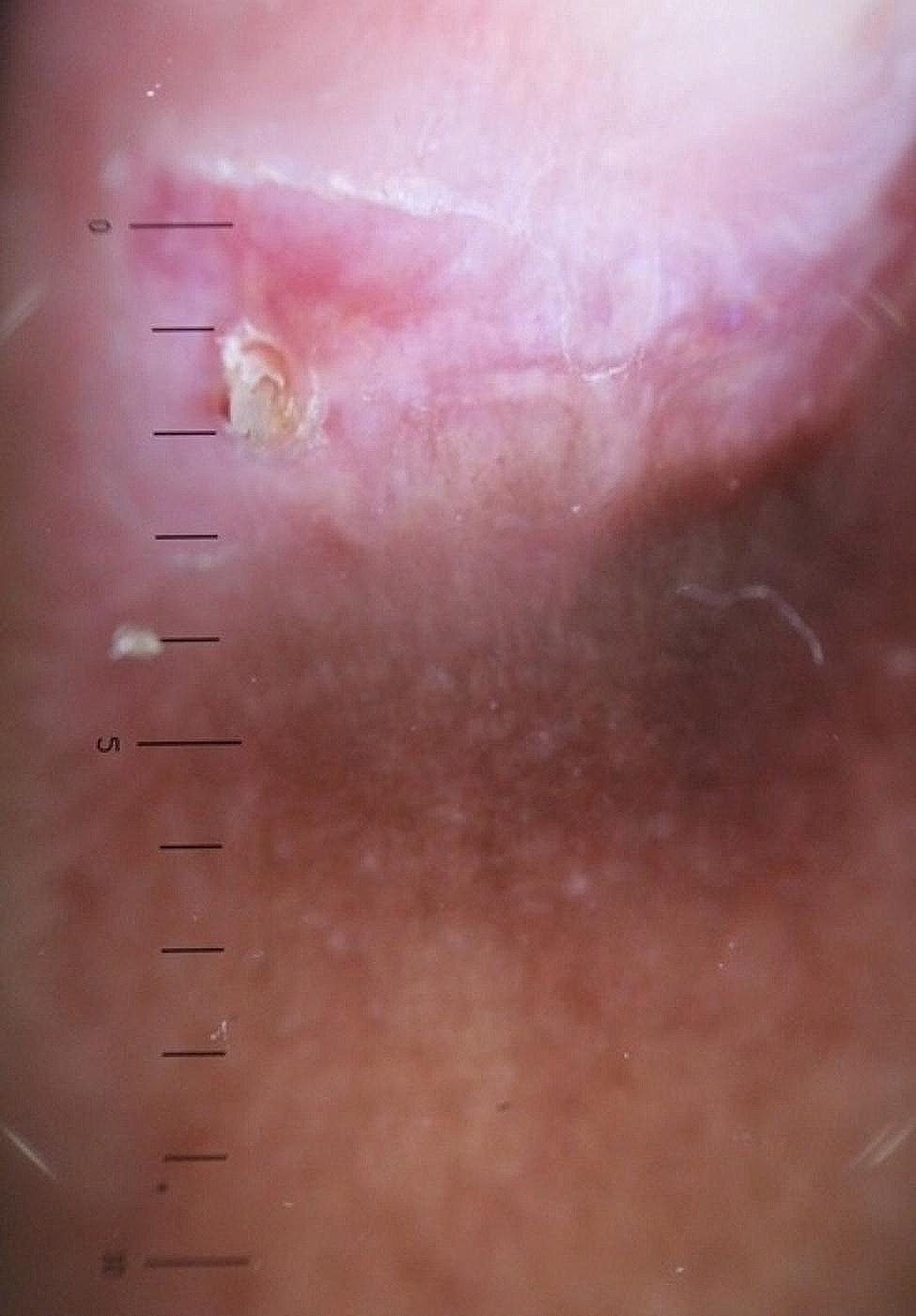
Anonychia in the nail of a leprosy patient (Heine DELTA 20 T Dermatoscope–polarizing light, ×10)

**Fig. 9:**
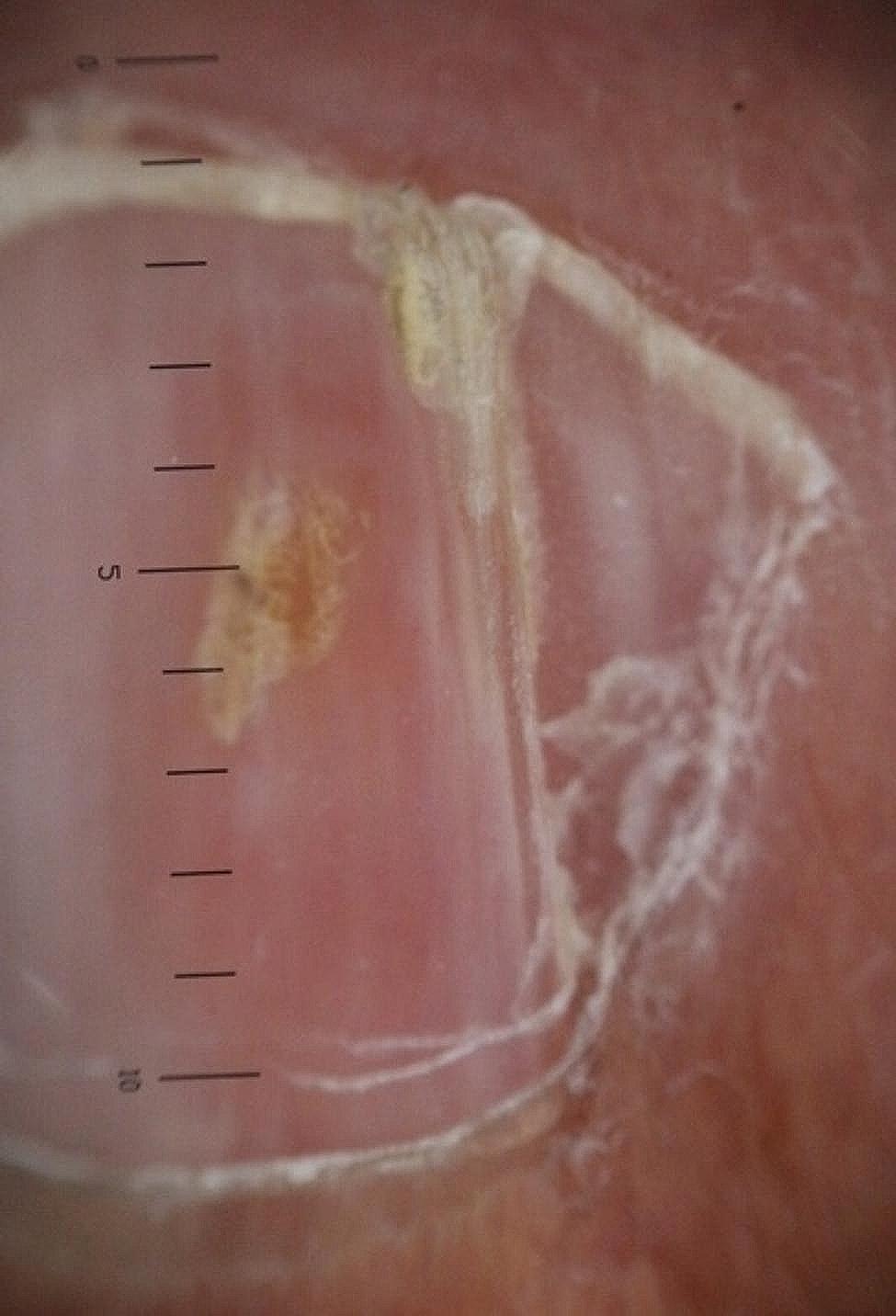
Onychorrexis in the nail of a leprosy patient (Heine DELTA 20 T Dermatoscope–polarizing light, ×10)

The longitudinal ridge, transverse line, onycholysis, longitudinal melanonychia, leukonychia, subungual hemorrhage, subungual hyperkeratosis, anonychia, and onychorexis were the dermoscopic findings discovered in this study. The longitudinal ridge (Fig. 1) was the most common dermoscopic feature found in this study, in 13 (13/15) patients, followed by the transverse line (11/15) (Fig. 2), onycholysis (8/15) (Fig. 3), longitudinal melanonychia (7/15) (Fig. 4), leukonychia (6/15) (Fig. 5), subungual hemorrhage (5/15) (Fig. 6), subungual hyperkeratosis (3/15) (Fig. 7), anonychia (2/15) (Fig. 8), and onychorexis (2/15) (Fig. 9). Among 300 nails, nail changes were found in 30 fingernails and 84 toenails. Longitudinal ridge (53.3%) was the most prevalent nail change in fingernails, followed by longitudinal melanonychia (26.6%), leukonychia (13.3%), subungual hyperkeratosis (3.3%), and anonychia (3.3%). The most common changes in toenails were transverse line (35.71%), followed by longitudinal ridge (20.2%), onycholysis (14.28%), subungual hemorrhage (7.1%), longitudinal melanonychia (5.9%), leukonychia (5.9%), anonychia (4.7%), subungual hyperkeratosis (3.5%), and onychorexis (2.3%) (Table 2).

**Table 2:** Dermoscopic features of nail in leprosy patients

Dermoscopic features	No. of patients (*n* = 15)			Total no. of nails involved	
	Total (proportion)	Hands (proportion)	Feet (proportion)	Fingers (*n* = 30) (%)	Toes (*n* = 84) (%)
Longitudinal ridge	13(13/15)	7(7/15)	6(6/15)	16 (53,3)	17 (2, 20)
Transverse line	11(11/15)	–	11(11/15)	–	30(35,71)
Onycholysis	9(9/15)	–	9(9/15)	–	12(14,28)
Longitudinal melanonychia	7(7/15)	5(5/15)	2(2/15)	8 (26,6)	5 (5, 9)
Leukonychia	6(6/15)	2(2/15)	4(4/15)	4 (3, 13)	5 (5, 9)
Subungual haemorrhage	5(5/15)	–	5(5/15)	–	6 (1, 7)
Subungual hyperkeratosis	3(3/15)	1(1/15)	2(2/15)	1 (3, 3)	3 (3, 5)
Anonychia	2(2/15)	1(1/15)	1(1/15)	1 (3, 3)	4 (4, 7)
Onychorexis	2(2/15)	–	2(2/15)	–	2 (2, 3)
Spoon nail	–	–	–	–	–
Ectopic nail	–	–	–	–	–
Onychauxis	–	–	–	–	–
Onychogryphosis	–	–	–	–	–
Pseudomacrolunula	–	–	–	–	–
Pterygium unguis	–	–	–	–	–
Pallor nails	–	–	–	–	–

According to the Ridley-Jopling classification, leprosy was classified into tuberculoid (TT), borderline tuberculoid (BT), borderline borderline (BB), borderline leprosy (BL), and lepromatous leprosy (LL). In this study, there was no patient with TT type; one patient was BT type; five patients had BB type; five patients had BL type; and three patients were LL type. There was one patient with pure neural leprosy (PNL). Longitudinal ridge, onycholysis, and longitudinal melanonychia were found in BT, BB, BL, LL, and PNL types. The transverse line was found in BT, BB, BL, and LL types. Longitudinal melanonychia was found in BB, BL, and PNL types. Leukonychia and subungual hemorrhage were found in BB, BL, and LL types. Subungual hyperkeratosis was found in BL, LL, and PNL types. Anonychia was found in the LL and PNL types. Onychorexis was found in the BL and PNL types (Table 3).

**Table 3:** Dermoscopic features of nail in leprosy patients based on type of leprosy and number of patients

Dermoscopic features	No. of patients		No. of patients		No. of patients		No. of patients		No. of patients		No. of patients	
	TT (*n* = 0)		BT (*n* = 1)		BB (*n* = 5)		BL (*n* = 5)		LL (*n* = 3)		PNL (*n* = 1)	
	Hands (proportion)	Feet (proportion)	Hands (proportion)	Feet (proportion)	Hands (proportion)	Feet (proportion)	Hands (proportion)	Feet (proportion)	Hands (proportion)	Feet (proportion)	Hands (proportion)	Feet (proportion)
Longitudinal ridge	–	–	–	1(1/15)	3(3/15)	4(4/15)	2(2/15)	2(2/15)	2(2/15)	2(2/15)	–	1(1/15)
Transverse line	–	–	–	1(1/15)	–	3(3/15)	–	5(5/15)	–	2(2/15)	–	–
Onycholysis	–	–	–	1(1/15)	–	3(3/15)	–	1(1/15)	–	1(1/15)	–	1(1/15)
Longitudinal melanonychia	–	–	–	–	3(3/15)	1(1/15)	1(1/15)	1(1/15)	–	–	1(1/15)	–
Leukonychia	–	–	–	–	2(2/15)	–	1(1/15)	1(1/15)	–	2(2/15)	–	–
Subungual haemorrhage	–	–	–	–	–	1(1/15)	–	2(2/15)	–	1(1/15)	–	–
Subungual hyperkeratosis	–	–	–	–	–	–	–	1(1/15)	1(1/15)	1(1/15)	–	1(1/15)
Anonychia	–	–	–	–	–	–	–	–	–	1(1/15)	1(1/15)	–
Onychorexis	–	–	–	–	–	–	–	1(1/15)	–	–	–	1(1/15)
Spoon nail	–	–	–	–	–	–	–	–	–	–	–	–
Ectopic nail	–	–	–	–	–	–	–	–	–	–	–	–
Onychauxis	–	–	–	–	–	–	–	–	–	–	–	–
Onychogryphosis	–	–	–	–	–	–	–	–	–	–	–	–
Pseudomacrolunula	–	–	–	–	–	–	–	–	–	–	–	–
Pterygium unguis	–	–	–	–	–	–	–	–	–	–	–	–
Pallor nails	–	–	–	–	–	–	–	–	–	–	–	–

TT: Tuberculoid leprosy, BT: Borderline tuberculoid leprosy, BB: Borderline leprosy, BL: Borderline lepromatous leprosy. LL: Lepromatous leprosy, PNL: Pure Neural Leprosy

## Discussion

According to data from the Ministry of Health of the Republic of Indonesia in 2018, new cases of leprosy in Indonesia were more prevalent in males (9.872 cases) compared to females (6.048 cases) [14]. In this study, males (13/15) had more nail changes in leprosy than females (2/15). Similar results were found in other studies by Kaur et al. [7] in India in 2002 and Theunuo et al. [6] in India in 2020. This might be due to their greater mobility, which increases the possibility of contact in males. Males are also more likely to seek therapy at a healthcare facility [15].

Our study showed a similar proportion between all age groups, but the highest proportion was found in the age group below 25 years old. This result was similar to a study by Rajput et al. [16] in India in 2020, which showed the most prevalent patients were in the age group below 25 years old. In another study by Singh et al. [17] in Nepal in 2019, the most affected age group was < 24 years old. This distribution shows that leprosy is more commonly found at a younger age, which may be due to the disease’s relatively long incubation period.

Only a few studies discuss the relationship between disease duration and nail changes in leprosy. In this study, the duration of disease in 14 (14/15) patients was below two years, and that of one (1/15) patient was more than two years. The result was different from a study by Kaur et al. [7] who mentioned that nail changes were more common in patients who had had leprosy for more than five years. The pattern of nail alterations did not change considerably as the disease duration increased beyond five years [7].

This study reported that nine (9/15) of the leprosy patients had nail changes in both fingers and toes, while six (6/15) patients had toe involvement only. There were no leprosy patients who had only finger involvement. A similar result was reported by Kaur et al. [7] who reported that 107 of 116 patients had both fingernails and toenails involved. Rajput et al. also reported a similar result: most leprosy patients had toenail involvement only rather than fingernail involvement only. Nail changes are more common on the toenails, presumably because the toenails are more exposed to trauma than the fingernails [16].

The longitudinal ridge (13/15) was the most common dermoscopic feature found in current studies. A similar result was found in a study by Theunuo et al. [6] that reported the longitudinal ridge as the most common dermoscopic feature (30/30) found in nails in leprosy patients. This study also showed longitudinal ridges in the BT, BB, BL, and LL types of leprosy, which is similar to a study conducted by Rajput et al. [16] Longitudinal ridges could be found in fingernails and toenails [7]. Longitudinal ridges were found not only in toenails but also in fingernails. This result was different from a study conducted by Kaur et al. [7] who reported longitudinal ridges only found in toenails. Longitudinal ridges are thought to occur due to trauma caused by decreased nerve function in leprosy patients; they were found on the nails of patients with various types of leprosy and are mostly found on the toenails [7, 16].

The transverse line (11/15) was found as one of the dermoscopic features in this study. A similar result was found in a study conducted by Theunuo et al. [6] who reported transverse lines (13/30) in leprosy patients. In this study, the transverse line was the most common dermoscopic feature found in toenails (35.71%) and mostly found in toenails in BL type of leprosy (53.8%). The transverse line in leprosy is presumably caused by a temporary pause in nail growth and may be related to treatment with dapsone and/or clofazimine [10].

Onycholysis was found in nine (9/15) patients in this study. These results were similar to those of studies conducted by Kaur et al., Rajput et al., and Theunuo et al. [6]. In this study, onycholysis was also found in most types of leprosy, mostly in the BB type (21.4%). The result was different from a study conducted by Rajput et al. who reported that onycholysis was mostly found in BT patients (7.7%). This study also reported that most onycholysis was found in toenails, similar to Ramos et al. [18] who reported that onycholysis was only found in toenails (49.3%). Onycholysis in leprosy was suspected to be caused by recurrent trauma [10], thus mostly found in toenails, which are more prone to trauma [16].

In the current study, longitudinal melanonychia was found in seven (7/15) patients. This was similar to other studies conducted by Kaur et al [7], Theunuo et al. [6], and Ramos et al. [18] In this study, longitudinal melanonychia was mostly found in fingernails (29.4%) in BB-type leprosy. This result was different from a study conducted by Rajput et al. [16] who reported longitudinal melanonychia was mostly found in the BL type of leprosy. Longitudinal melanonychia is a line of pigmentation that extends from the lunula to the outer border of the nail bed. In leprosy, these bands presumably develop due to repeated trauma, thus stimulating melanocytes in the nail matrix [10].

Leukonychia was found in six (6/15) patients during this study. The result was similar to that of Theunuo et al. [6] who reported leukonychia (46.7%) in leprosy patients. Leukonychia is a white staining of the nail and can be found in leprosy patients [6, 10]. In leprosy, leukonychia might result from trauma due to a decrease in nerve function [10].

Subungual hemorrhage was found in five (5/15) patients and was only present in toenails. The result was similar to the study conducted by Ramos et al. [18]. Subungual hemorrhage was also found in BB, BL, and LL types of leprosy. To date, there have been no studies explaining the relationship between subungual hemorrhage and the type of leprosy. Trauma may cause subungual hemorrhage leading to brown-black pigmentation in leprosy patients’ nails [10].

Subungual hyperkeratosis was found in three (3/15) patients in this study. This result was similar to the study conducted by Rajput et al. [16], Kaur et al. [7], and Theunuo et al. [6]. This study also found that subungual hyperkeratosis was found in BL, LL, and PNL types. This result was different from Rajput et al. [16], who reported that subungual hyperkeratosis was found in all types of leprosy. Subungual hyperkeratosis in leprosy has been speculated to be caused by the adverse effect of clofazimine therapy, but the mechanism is still unknown [21].

In the current study, anonychia was found in two (2/15) patients with LL and PNL types of leprosy. The result was similar to a study conducted by Rajput et al. [16]. Anonychia in leprosy may develop when the underlying bone is either hypoplastic or entirely missing due to the resorption of the distal phalanx [10].

Onychorrhexis was found in two (2/15) patients during the study and was only found in toenails. This result was similar to Rajput et al., who reported onychorrhexis in leprosy patients and mostly found in toenails. Theunuo et al. [6] also reported onychorrhexis, found in fingernails. Onychorrhexis in leprosy may result from a brittle nail plate and may be related to longstanding leprosy [10].

Nail changes in leprosy are not only cosmetic, influencing social contact, but also functional, affecting day-to-day functioning. Thus, being aware of nail changes could help in diagnosis.

## Conclusion

In conclusion, nail changes in leprosy are present and have a wide variety of clinical appearances. Nail changes could result from many different processes. Dermoscopy is a simple method to observe nail changes in leprosy.

## Data Availability

Data supporting this study’s findings are available from the corresponding author upon reasonable request.
